# Surface modification of zirconia or lithium disilicate-reinforced glass ceramic by laser texturing to increase the adhesion of prosthetic surfaces to resin cements: an integrative review

**DOI:** 10.1007/s00784-023-05016-z

**Published:** 2023-04-17

**Authors:** Júlio C. M. Souza, Angelo Raffaele-Esposito, Oscar Carvalho, Filipe Silva, Mutlu Özcan, Bruno Henriques

**Affiliations:** 1 Center for Microelectromechanical Systems (CMEMS), University of Minho, Campus de Azurém, 4800-058 Guimarães, Portugal; 2 LABBELS Associate Laboratory, University of Minho, 4710-057 Guimarães, Portugal; 3 University Institute of Health Sciences (IUCS), CESPU, Gandra, PRD 4585-116 Portugal; 4 Division of Dental Biomaterials, Center of Dental Medicine, Clinic of Reconstructive Dentistry, University of Zurich, 8032 Zurich, Switzerland; 5 Ceramic and Composite Materials Research Group (CERMAT), Department of Mechanical Engineering (EMC), Federal University of Santa Catarina (UFSC), Florianópolis, SC 88040-900 Brazil

**Keywords:** Lithium disilicate, Zirconia, Laser, Bond strength, Resin cement

## Abstract

**Objective:**

The purpose of this study was to perform an integrative review on laser texturing the inner surface of lithium disilicate-reinforced glass ceramic or zirconia to increase their bond strength to resin-matrix cements.

**Materials and method:**

A bibliographic review was performed on PubMed using the following search terms: “zirconia” OR “lithium disilicate” AND “laser” AND “surface” OR “roughness” AND “bond strength” AND “luting agent” OR “resin cement.” Studies published in English language until March 15, 2023, were selected regarding the purpose of this study.

**Results:**

A total of fifty-six studies were identified althoug thirteen studies were selected. The findings revealed that zirconia surfaces were significantly modified after laser irradiation resulting in macro-scale aligned retentive regions with depth values ranging from 50 to 120 µm. Average roughness values of laser-textured zirconia by Er,Cr:YSGG laser (~ 0.83 µm) were quite similar when compared to grit-blasted zirconia surfaces (~ 0.9 µm) although roughness increased up to 2.4 µm depending on the laser type and parameters. Lithium disilicate-reinforced glass ceramics textured with Er:YAG revealed an average roughness of around 3.5 µm while surfaces textured using Nd:YAG laser revealed an average roughness of 2.69 µm; that was quite similar to the roughness values recorded for etched surfaces (2.64 µm). The shear bond strength (SBS) values of zirconia surfaces textured on Nd:YVO_4_ laser irradiation were slightly higher (~ 33.5 MPa) than those recorded for grit-blasted zirconia surfaces (28 MPa). Laser-textured zirconia surfaces on CO_2_ laser revealed higher SBS values (18.1 ±0.8 MPa) than those (9.1 ± 0.56 MPa) recorded for untreated zirconia surfaces. On lithium disilicate-reinforced glass ceramics, higher SBS values to resin-matrix cements were recorded for specimens textured with a combination of fractional CO_2_ laser irradiation and HF acid etching (~ 22–24 MPa) when compared with grit-blasted specimens (12.2 MPa). Another study revealed SBS values at around 27.5 MPa for Er:YAG-textured lithium disilicate-reinforced glass ceramics to resin-matrix cements.

**Conclusions:**

The laser irradiation at high power increases the roughness of the inner surface of lithium disilicate-reinforced glass ceramic or zirconia leading to an enhanced bond strength to resin-matrix cements. Thus, the laser type and irradiation parameters can be adjusted to enhance the macro- and micro-scale retention of zirconia and glass ceramic surfaces to resin-matrix cements.

**Clinical relevance:**

Alternative methods for surface modification of lithium disilicate-reinforced glass ceramic and zirconia surfaces have been assessed to provide proper morphological aspects for enhanced adhesion to resin-matrix cements. An increase in the bond strength of glass ceramics or zirconia to resin-matrix cements can improve the long-term performance of cemented prosthetic structures in the oral cavity.

## Introduction

Nowadays, the use of polycrystalline ceramics and glass ceramics has been increasingly preferred by clinicians and patients considering esthetic outcomes [1, 2]. Lithium disilicate-reinforced glass ceramics and polycrystalline ceramics such as zirconia are the first choice for manufacturing veneer, crown, inlay, and onlay restorations over teeth or dental implant abutments [1, 2]. The surface modification of dentin or enamel is carried out by acid etching with 37% H_3_PO_4_ acid followed by conditioning with methacrylate-based adhesives for adhesion to glass ceramics or zirconia. Titanium abutment base can also be modified by grit blasting with alumina particles (Al_2_O_3_) followed by acid etching with (5–10%) HF acid and then conditioning with silane and methacrylate-based adhesives [1, 3] (1,9). In the same way, surfaces of lithium disilicate-reinforced glass ceramics can also be modified by acid etching with (5–10%) HF acid providing a standard rough surface for adhesion to adhesive systems and resin-matrix cements. Thus, the roughness of enamel, dentin, glass ceramics, and titanium surfaces can be increased by acid etching that increase the adhesion area for mechanical interlocking to adhesives and resin-matrix cements [1–4]. However, acid etching with HF or H_3_PO_4_ acids cannot alter surfaces of polycrystalline ceramics such as zirconia considering a highly chemical stability of the ceramic [5, 6]. Zirconia has well-reported enhanced mechanical properties although the surface modification using traditional methods for further adhesion to resin-matrix cements is a major disadvantage [2, 5–9]. Therefore, alternative surface modification methods must be studied to enhance the zirconia bonding to resin-matrix cements [6, 7, 10].

The long-term clinical success of ceramic-based restorations is strongly dependent on their retention to teeth or abutment substrates by using adhesive systems and resin-matrix cements [1, 2, 7]. Traditional methods of surface modification such as grit blasting and acid etching have limited capability to achieve optimum morphological aspects and roughness over zirconia for mechanical interlocking of adhesive systems and resin-matrix cements [8–10]. Also, grit blasting with Al_2_O_3_ or SiO_2_ particles shows limitations considering limited morphological aspects, contamination with remnant abrasive particles, and risks of cracks’ propagation [5]. The combination of different traditional surface modification techniques has been studied and associated with novel methods such as laser-assisted approaches [6, 7, 10].

The laser-assisted approaches already have many applications in oral pathology, endodontics, operative dentistry, and prosthodontics [5, 6, 10, 11]. The surface modification of implants and prosthetic structures by high intensity laser irradiation has gathered attention to improve roughness and wettability for adhesion to resin-matrix cements [7, 10, 12–15]. Currently, different morphological surface features (i.e., micro-grooves, cross-lines, pits, valleys, and peaks) can be produced on ceramics and glass ceramics by using different laser intensity, type, time, and frequency [7, 10, 12–17]. Previous studies have reported the surface modification of zirconia leading to an increased roughness after short (mili- to nano-seconds) or ultra-short (pico- to femtoseconds) periods of time (pulses) using different types of lasers such as Nd:YAG, Er:YAG, CO_2_, Er,Cr:YSGG, and Nd:YVO_4_ [7, 10, 12–19]. However, zirconia is susceptible to defects such as cracks depending on the mode and intensity of the laser irradiation. Minimal surrounding defects and contaminants have been reported after surface modification of zirconia by using ultra-short pulsed lasers such as pico- and femtosecond lasers [7, 10, 13]. Also, a minimal tetragonal-to-monoclinic phase transformation was detected in zirconia after irradiation with ultra-short laser pulses [19]. Thus, further studies are required to validate adequate parameters for surface modification of zirconia and glass ceramics by laser-assisted approaches to improve their bond strength to resin-matrix cements.

The main aim of the present study was to carry out an integrative review on laser texturing the inner surface of zirconia in comparison to lithium disilicate-reinforced glass ceramics used for enhanced bonding of veneer and crowns to resin-matrix cements. It was hypothesized that the laser irradiation at high power increases the roughness of the inner surface of zirconia or lithium disilicate-reinforced glass ceramics leading to an enhanced bond strength to resin-matrix cements.

## Method

### Information sources and search strategy

A bibliographic review was performed on PubMed (via National Library of Medicine) taking into account such database includes the major journals in the field of dentistry and biomaterials. The present method was performed in accordance with the search strategy applied in previous studies on integrative or systematic reviews [2, 6, 11, 20–25]. The following search terms were applied: “zirconia” OR “lithium disilicate” AND “laser” AND “surface” OR “roughness” AND “bond strength” AND “luting agent” OR “resin cement.” At first, combination of three or four key terms was used to find relevant studies.

Also, a hand search was performed on the reference lists of all primary sources and eligible studies of this integrative review for further relevant publications. The inclusion criteria encompassed articles published in the English language, from January 2012 until March 15, 2023, reporting the effects of the laser irradiation on the surface modification of lithium disilicate-reinforced glass ceramic or zirconia veneers and their adhesion to resin-matrix cements. The eligibility inclusion criteria used for article searches also involved: in vitro studies, meta-analyses, randomized controlled trials, animal assays, and prospective cohort studies. The exclusion criteria were the following: papers without abstract, case report with short follow-up period, and articles assessing only traditional methods of surface modification. Studies based on publication date were not restricted during the search process.

### Study selection and data collection process

At first, studies were examined for relevance by title, and the abstracts were assessed for those studies which were not excluded at this step. Two of the authors (JCMS, ARE) individually analyzed the titles and abstracts of the retrieved potentially relevant articles meeting the inclusion criteria. The total of the studies was compiled for each combination of key terms, and therefore, the duplicates were removed using Mendeley citation manager. The second step comprised the evaluation of the abstracts following the eligibility criteria. Selected studies were independently read and analyzed concerning the purpose of this study. At last, the eligible articles received a study nomenclature label, combining first author names and year of publication. The following variables were collected for this review: authors’ names, journal, publication year, aims, materials, laser parameters, roughness, and adhesion. PICO question was adjusted to the issue where “P” was related to the patients or specimens while “I” referred to the methods of analyses while “C” was related to comparison of findings and “O” to the main outcomes. Data of the reports were harvested directly into a specific data collection form to avoid multiple data recording considering multiple reports within the same study (e.g., reports with different set-ups). This evaluation was individually carried out by two researchers, followed by a joint discussion to select the relevant studies.

## Results

**Fig. 1:**
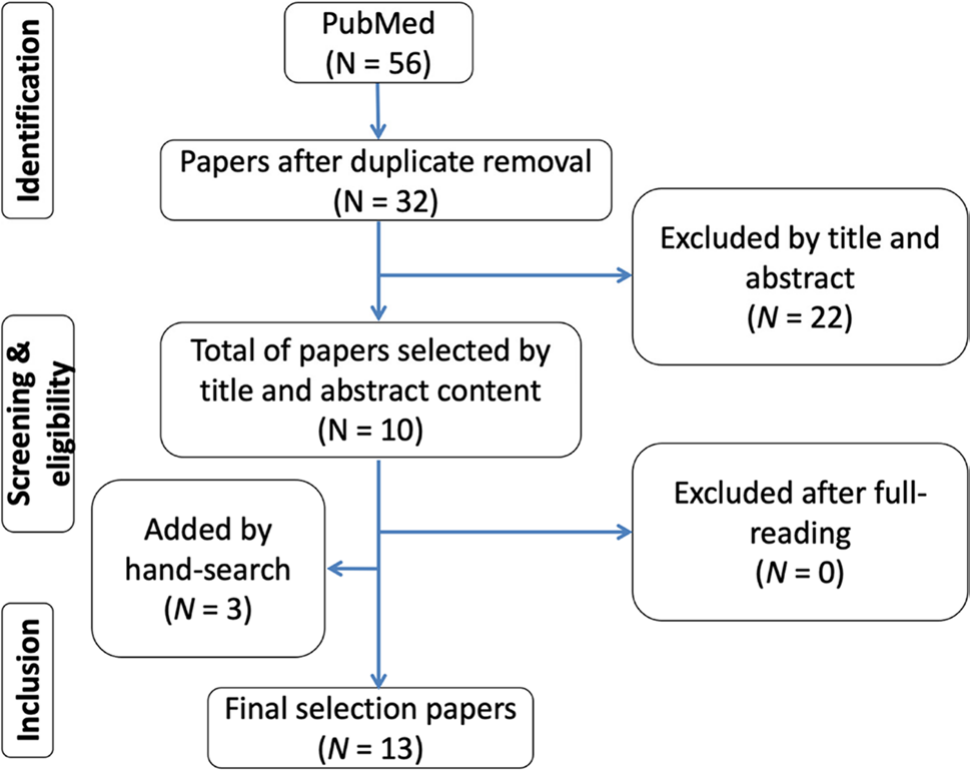
Study selection flowchart

The search of articles on PubMed identified a total of 56 studies and duplicates were removed considering the initial combination of key terms, as seen in Fig. 1. A total of 22 studies was discarded considering that they did not meet the inclusion criteria. The remnant 10 studies were full read and selected. Three studies were added by hand search since they were considered as relevant regarding information on laser parameters, methods, and main outcomes.

Of the 13 selected studies, yttria-stabilized tetragonal zirconia polycrystals (Y-TZP) were assessed by 9 studies [8, 9, 26–32] although ceria-stabilized tetragonal zirconia polycrystals (Ce-TZP) were also assessed by one study [27]. The effects of laser irradiation on lithium disilicate-reinforced glass ceramic were investigated by four studies [33–36]. The following types of laser were assessed by the selected studies: Er,Cr:YSGG [9, 30], Er:YAG [33, 34, 36], Nd:YAG [34], CO_2_ [8,32,35,], and Nd:YVO_4_ [27]. The laser irradiation parameters varied among the studies as seen in Table 1. For instance, the power of the Er,Cr:YSGG laser irradiation ranged from 1 up to 6 W [9, 30] while Er:YAG laser was assessed at power intensity range between 4 and 10 W [33–35]. The power assessed for CO_2_ laser ranged from 3 up to 20 W [8, 32, 35] while the power of Nd:YAG laser was assessed at 2.5 W [34]. After laser irradiation, roughness values were recorded by four studies [26, 29, 32, 34] although the shear bond strength (SBS) of laser-textured zirconia to resin-matrix cements was recorded by each one of the selected studies.

**Table 1:** Data retrieved from the selected studies

Authors (year), country	Study design and follow-up	Ceramic	Resin-matrix cement	Laser type	Laser parameters	Roughness (µm)	Main outcomes
Toyoda et al. (2022), Japan	In vitro - Shear bond test at 0.5 mm/min using a universal machine (Autograph, AGS-J, Shimadzu, Japan) - Thermal cycling consisted at 5 °C for 1 min and 55 °C for 1 min - Scanning electron microscopy (SEM; JSM-6330F, JEOL, Tokyo, Japan) - Profilometry as the arithmetic mean height of the surface (Sa) and developed interfacial area ratio (Sdr)	ZrO_2_ (97wt%), Y_2_O_3_ (3wt%) (Daiichi Kigenso, Japan)	1) Powder: PMMA, TiO_2_; liquid: 4-META, MMA; catalyst: TBB (Super bond C&B™, Sun Medical, Japan) 2) Bis-GMA, TEGDMA, silanated barium glass filler, silanated fluoroalminosilicate glass filler, colloidal silica, surface treated aluminum oxide filler, hydrophobic aromatic dimethacrylate, hydrophilic aliphatic dimethacrylate, dl-camphorquinone, initiators, accelerators, pigments (Panavia V5™, Kuraray Noritake, Japan) Priming agent	Ytterbium fiber laser system (MD-F3000, Keyence, Osaka, Japan)	Power output of 24 W and pulse frequency of 60 kHz for 6.6 s of laser irradiation time	Arithmetic mean height (Sa): Untreated: 0.19 ± 0.06 Grit-blasted with (Al_2_O_3_): 0.55 ± 0.05 Laser-textured: 2.39 ± 0.02	Shear bond strength mean values (MPa): 1) Super bond C&B™: Untreated before thermal cycling: 10 Untreated after thermal cycling: 10 Grit-blasted before thermal cycling: 13 Grit-blasted after cycling: 23 Laser-textured before thermal cycling: 14.5 Laser-textured after thermal cycling: 26 2) Panavia V5 ™: Untreated before thermal cycling: 11 Untreated after thermal cycling: 0 Grit-blasted before thermal cycling: 14.5 Grit-blasted after thermal cycling: 2 Laser-textured before thermal cycling: 12 Laser-textured after cycling: 10 No significant difference between grit-blasted and laser-textured
Uno & Ishigami (2022), Japan	In vitro Scanning electron microscopy (SEM) X-ray diffraction (XRD) Shear bond test Optical profilometry	ZrO_2_, HfO_2,_ (5.15wt%) Y_2_O_3_, (0.25wt%) Al_2_O_3_, (≤ 0.04 wt%) Na2O, (≤ 0.01 wt%) Fe_2_O_3_ (TZ-3YSB-E Tosoh, Tokyo, Japan)	Adhesive resin cement (SA Multi, Kuraray Noritake Dental) A Pen Cure 2000 VL-10 (2,000 mW/cm^2^) (Morita, Kyoto, Japan) was used to irradiate the DC core with light for 20 s under a weight of 9.8 N	CO_2_ laser, wavelength of 9.36 ± 0.05 μm (Diamond J-5 Series™, Coherent, Santa Clara, CA, USA)	Output of 0.62 W, a beam waist diameter at 1/e2 of 7.0 ± 1.0 mm, and a pulse frequency of 200 kHz Radio frequency excitation pulse width ranged from 2 to 800 μs 25 lines of cone-shaped dents in the vertical and horizontal directions were shaped as the spots	Ra roughness (μm): Machined: 0.3 Grit-blasted: 0.58 Laser-textured: 0.8 Grit-blasted plus Laser-textured: 0.95	The shear bond strength of grit-blasted or laser-textured zirconia to resin cement was 2.3 times higher than that for untreated zirconia No significant difference was detected between grit-blasted and laser-textured zirconia
Fornaini et al. (2021), France and Italy	In vitro - Optical profilometry (Talysurf CCI green light, Taylor Hobson, UK) - Shear bond strength testing (Erichsen, Wuppertal, Germany) at a 0.05 mm/s crosshead speed	Zirconia (DDBioZ, Dental Direkt GmbH, Spenge, Germany); ZrO_2_ + HfO_2_ + Y_2_O_3_ (≥ 99wt%), Al_2_O_3_ (0.25wt%), and other oxides (< 0.1wt%)	Primer (Zirconia Prime, DenMat, USA) Flowable resin-matrix composite (Tetric EvoFlow™, Ivoclar Vivadent, Germany)	1070 nm Yb-doped pulsed fiber laser (AREX 20, Datalogic, Italy)	1070 nm Yb-doped pulsed fiber laser with a maximum average output power of 20 W, a fixed pulse duration of 100 ns, and a repetition rate in the range 20–100 kHz	-	- The highest forces were recorded for laser-treated surfaces (132.5 ± 61.35 N) than those for non-treated ones (105.7 ± 56.1 N). However, the difference was not considered statistically significant (*p* = 0.2155) Lines depth: 10 µm Depth at 10 W: 100 µm Depth at 20 W: 120 µm
Turker et al. (2020), Turkey	In vitro - The pull-out testing at 0.5 mm/min crosshead speed (Model AG-50 kNG, Shimadzu) - Surfaces were examined under a stereomicroscope (SZH10, Olympus)	Zirconia: Y-TZP; ZrO_2_ (97wt%), Y_2_O_3_ (3wt%)	A dual-cure self-adhesive resin cement (RelyX U200™, 3 M ESPE)	ER,CR:YSGG 1.5 or 3 W	1) Power of 1.5 W at 20 Hz frequency and 80% air and 25% water at a distance of 1 mm for 30 s 2) Power of 3 W at 20 Hz frequency and 80% air and 25% water at a distance of 1 mm for 30 s	-	- The mean bond strength values of titanium were higher than those of the zirconia - For zirconia, all four treatment groups (two lasers, grit blasting, and tribochemical silica coating) significantly differed from the control group, although the treatment groups were not statistically different from each other
Iwaguro et al. (2019), Japan and Taiwan	In vitro - SEM (SE-8000, Keyence Corp., Osaka, Japan) at × 500 magnification - Shear bond test at a crosshead speed of 0.5 mm/min using a universal testing machine (AGS-5kNJ, Shimadzu, Kyoto, Japan) - Groups were submitted on thermocycling (Thermal Cycler, Nissin Seiki Co. Ltd., Hiroshima, Japan) at 4 °C and 60 °C in water (dwell time per water bath for 1 min) for 20,000 cycles	Zirconia: 1) Y-TZP; ZrO_2_ (97wt%), Y_2_O_3_ (3wt%) (Tosoh Corp., Japan) 2) Ce-TZP/A (ZrO_2_, Al_2_O_3_, CeO_2_, Yamakin Co., Ltd., Japan) Porcelain: Vintage ZR, opaque liner A3O and body A3B = aluminosilicate glass, glycerin, propylene glycol (Shofu Inc., Japan)	Gradia™ (GC Corp., Tokyo, Japan): - Foundation opaque: UDMA, SiO_2_ - Opaque A3: UDMA, SiO_2_ - Dentin A3: UDMA, SiO_2_, filler, grass powder Primer: Clearfil Ceramic Primer Plus™ with MDP, ethyl alcohol (Kuraray Noritake Dental Inc., Tokyo, Japan)	Nd:YVO_4_, CNC laser machine (LASER-TEC 40, DMG MORI, Japan)	Frequency of 70 kHz, wavelength of 1,065 nm, distance from the surface of 60 mm, and angle of incidence of 90°		Shear bond strength mean values (MPa): 1) Y-TZP: Alumina-blasted: 28.9 Alumina-blasted after thermal cycling: 15.5 Laser-textured with microslits at 50 μm: 28.6 Laser-textured after cycling with microslits at 50 μm: 33.1 Laser-textured with microslits at 75 μm: 33.5 Laser-textured after cycling com microslits at 75 μm: 31.5 Laser-textured with microslits at 100 μm: 29.2 Laser-textured after cycling com microslits at 100 μm: 33.5 2) Ce-TZP/A: Alumina-blasted: 25.6 Alumina-blasted after thermal cycling: 16.6 Laser-textured with microslits at 50 μm: 27.1 Laser-textured after cycling with microslits at 50 μm: 34.5 Laser-textured with microslits at 75 μm: 26.6 Laser-textured after cycling with microslits at 75 μm: 32.9 Laser-textured with microslits at 100 μm: 27.6 Laser-textured with microslits at 100 μm after thermal cycling: 32 The predominant failure on laser-textured surfaces was a cohesive fracture
Akpinar et al., (2015), Turkey	In vitro Shear bond test at a crosshead speed of 1 mm/min using a universal testing machine (AGS-X, Shimadzu, Kyoto, Japan) Scanning electron microscopy (SEM)	Y-TZP (Zirkonzahn USA Inc., USA)	A dual-cure self-adhesive resin cement (RelyX U200™, 3 M ESPE)	Femtosecond amplifier laser pulse, wavelength of 810 nm (Quantronix Integra-C-2.5, NY, USA)	Output with 750 mW per pulse and with 90 fs and 2 kHz repetition rate. Laser beam was delivered by laser marker (Q-Mark, Quantronix, NY, USA). Scan work plane for 30 mm/s scanning speed		The contact surface area (μm^2^) between zirconia and resin cement increase: 90 (3848), 75 (3982) 60 (4442), 45 (5447) The laser beam was applied to a surface with a 45° angle which resulted in significantly higher SBS (18.2 ± 1.43 MPa) than other groups (at 90° angulation (10.79 ± 1.8 MPa), at 75° (13.48 ± 1.2 MPa) and at 60° (15.85 ± 0.81 MPa)
Kirmali et al. (2015), Turkey	In vitro - All specimens were mounted on metallic stubs, gold sputter coated (Polaron Range SC 7620; Quorum Technology, Newhaven, UK) - Shear bond testing (Lloyd LF Plus; Ametek Inc., UK) - Roughness (Ra, lm) was determined with a profilometer (Mitutoyo Surftest SJ-301, Japan)	Pre-sintered Y-TZP zirconia cylinders (Noritake, Japan) ZrO_2_ (97wt%), Y_2_O_3_ (3wt%) Sintering at 1500 °C for 8 h in a Zyrcomat (VITA Zahnfabrik, Germany) sintering furnace	Resin-matrix cement (unknown)	Er,Cr:YSGG laser irradiation	Irradiation at 2.78 µm wavelength. Optical fiber of the laser (600 µm diameter, 6 mm length) was placed at a distance of 10 mm Pulse duration from 140 to 200 µs with a repetition rate of 20 Hz (pulses/sec) and pulsed laser-powered hydrokinetics, the output power from 0.25 to 6.0 W. The energy parameters at 1, 2, 3, 4, 5, and 6 W and water/air flow of 55% and 65% were used continuously during the irradiation for 20 s	Control: 0.75 Grit-blasted: 0.90 1 W laser: 0.79 2 W laser: 0.82 3 W laser: 0.83 µm 4 W laser: 0.84 µm 5 W laser: 0.84 µm 6 W laser: 0.83 µm	- Surfaces treated by grit blasting became rough and irregular and showed depression areas - 43.75% fracture occurred at the veneer ceramic/zirconia interface (adhesive failure) Shear bond strength (MPa) to resin cement: - Control: 11.31 - Air abrasion: 23.31 - 1W laser: 13.2 - 2W laser: 13.65 - 3W laser: 16.83 - 4W laser: 18.50 - 5W laser: 21. 69 - 6W laser: 22.99
Murthy et al. (2014), India	In vitro - The shear bond strength testing to resin-matrix cement - Laser-treated surfaces compared to grit-blasted with alumina (110 or 250 μm)	Zirconia, CEREC (ZrO_2_) (Cortis-YZ, Sirona Dental GmbH Bensheim, Germany)	Resin cement block of 0.5 mm (unknown)	Surgical CO_2_ laser radiation (Smart US 20D, CO_2_ laser, Deka Florence, Italy)	Laser energy in a pulse mode with wavelength of 10.6 mm, a pulse repetition rate of 1000 Hz, and a pulse duration of 160 ms at an average power setting of 3 w at 1 mm away from the surface	-	- Laser-treated surfaces revealed the highest shear bond strength values (18.1 ± 0.8 MPa) while lowest values were recorded for control group (9.1 ± 0.56 MPa) - There were no significant differences between the control and grit-blasted groups
Ergun-Kunt et al. (2021), Turkey	In vitro - Shear bond testing (Micro Tensile Tester; BISCO Dental Products, USA) - Optical microscopy (Kaps ENT SOM Microscope, Germany) - SEM analyses (JSM-7001F, JEOL, Japan)	Lithium disilicate-reinforced glass ceramic: IPS e.max Press™ (Ivoclar-Vivadent, Liechtenstein)	Resin-matrix composite: Tetric N-Ceram™ (Ivoclar-Vivadent, Liechtenstein) Silane coupling agent: 3-glycidoxypropyltrimethoxysilane (Ivoclar-Vivadent, Liechtenstein)	Er:YAG laser	Frequency of 20 Hz, within a long pulse of 5 W, and a energy of 250 mJ for 30 s	-	- Surfaces treated with silane followed by laser irradiation had the highest mean bond strength values to resin composites (27.84) - Surfaces grit-blasted with Al_2_O_3_ particles showed the lowest mean bond strength to the resin composite (15.62 MPa) - A silane heat treatment by Er:YAG laser resulted in deterioration and contamination on the ceramic
Feitosa et al. (2017), Brazil	In vitro - Bond strength testing (model DI-1000; EMIC, Brazil) - Optical profilometry was for the arithmetic mean value of surface roughness (Ra) - SEM analyses (Inspect S50, FEI Company, USA) at different magnifications	Lithium disilicate-reinforced glass ceramic: IPS e.max Press–LTD3™ (Ivoclar-Vivadent, Liechtenstein); SiO_2_, Li_2_O, K_2_O, MgO, ZnO, Al_2_O_3_, P_2_O_5_, and other oxides	Dual-cure resin cement; = Bis-GMA, urethane dimethacrylate, triethylene glycol dimethacrylate. Barium glass, ytterbium trifluoride, Ba-Al-fluorosilicate glass, spheroid mixed, oxide, catalysts, stabilizers, pigment (Variolink II™ (base & catalyst, Ivoclar-Vivadent, Liechtenstein) Silane: Alcohol solution of silane methacrylate, phosphoric acid methacrylate and sulfide methacrylate (Monobond Plus™ (Ivoclar-Vivadent, Liechtenstein)	1) Er:YAG laser (Key Laser 3; KaVo Kerr, USA) 2) Nd:YAG laser (PulseMaster 600 IQ; American Dental Technologies Inc., USA)	1) Er:YAG laser: 200 mJ energy, using a pulse repetition rate set at 10 pps, 2.94 μm wavelength and at 12 mm away from the s surface with water spray cooling (5 s) 2) Nd:YAG laser: 120 mJ energy. The pulse repetition rate was set at 15 pps, and a 320 μm diameter laser optical fiber was placed in contact with the surface for 1 min without water spray Each irradiated area was etched with HF for 60 s	(R_a_ roughness): Control (only HF): 2.64 (center) and 1.39 (periphery) Er:YAG: 3.48 (center) and 2.14 (periphery) Er:YAG + graphite: 1.29 (center) and 1.16 (periphery) Nd:YAG: 2.69 (center) and 1.46 (periphery) Nd:YAG + graphite: 0.91 (center) and 0.86 (periphery)	- Er:YAG laser group showed the highest bond strength (27.5 ± 7.1) - Both the factors “laser” (*p* = 0.00) and “graphite coating” (*p* = 0.00) significantly affected the bond strength of the resin-matrix cement to the lithium disilicate ceramic. The interaction of those two factors was not statistically significant (*p* = 0.059)
Ahrari et al. (2017), Iran	In vitro - Optical microscopy (Dino Lite Pro, AnMo Electronics Corp., Taiwan, ROC) at × 20 magnification for fracture inspection - Shear bond strength testing (Santam, Model STM-20, Iran)	Lithium disilicate-reinforced glass ceramic: IPS e.max CAD™ (Ivoclar-Vivadent, Liechtenstein)	A dual‐cure self-adhesive resin luting cement (Clearfil SA Luting™, Kuraray Noritake Dental Inc., Japan) Silane coupling agent (Silane Bond Enhancer, Pulpdent Corp., USA)	Fractional CO_2_ laser (a wavelength of 10.6 μm; Lutronic Inc., Princeton Junction, NJ, USA)	Two powers: 1) The frequency of 200 Hz (pulse per second) and irradiation time of 10 s. The power and pulse energy were 10 W and 14 mJ, respectively. The pulse duration was 1.75 ms, and the energy delivered was approximately 28 J 2) The power of 20 W and pulse energy of 10 mJ. The pulse duration was 0.58 ms, and the energy delivered was approximately 24 J	-	- The highest bond strength values were recorded for specimens treated with a combination of fractional CO_2_ laser irradiation and HF acid etching (22.4 and 24.3 MPa for groups 5 and 6, respectively). Grit-blasted specimens exhibited the lowest shear bond strength (12.2 MPa) - The adhesive failure was the main type of fracture followed by mixed fracture Shear bond strength (MPa) of resin cement to lithium disilicate specimens CO_2_ laser (20 W/10 mJ) + acid etching: 24.3 ± 7.2 CO_2_ laser (10 W/14 mJ) + acid etching: 22.4 ± 4.6 CO_2_ laser (20 W/10 mJ): 13.8 ± 2.3 CO_2_ laser (10 W/14 mJ): 13.8 ± 3.0
Yavuz et al. (2012), Turkey	In vitro Shear bond test at a crosshead speed of 0.5 mm/min using a universal testing machine (TSTM 02,500, Elista Ltd. Sti, Istanbul, Turkey) Optical microscopy (Olympus CX41, X40, Japan) One additional specimen from each group was evaluated by atomic force microscopy (AFM; NTEGRA Solaris, NTMDT, Russia)	Lithium disilicate-reinforced glass ceramic: IPS Empress 2 (Ivoclar-Vivadent, Liechtenstein) Specimens subjected to airborne particle abrasion with 50 μm Al_2_O_3_ particles at a pressure of 2 bar (Korox, Bego, Bremen, Germany)	Bis-GMA, TEGDMA, silanated barium glass filler, silanated fluoroalminosilicate glass filler, amorphous silica, surface treated aluminum oxide filler, hydrophobic aromatic dimethacrylate, hydrophilic aliphatic dimethacrylate, dl-camphorquinone, initiators, accelerators, pigments (Panavia 2.0™, Kuraray Noritake, Japan)	Er:YAG laser (Fotona, At Fidelis, Ljubljana, Slovenia)	Distance at 1 mm, 500 mJ (pulse energy), 37.68 J/cm^2^ (energy density), 20 Hz (pulses/s), SP mode (100 μs pulse length), 10 W Handheld scanned within a water cooling, R14 handpiece	AFM images revealed rough morphological aspect for grit-blasted surfaces	Shear bond strength (MPa): Grit-blasted: 6.1 HF etched: 6.4 Laser-textured: 4.5 Grit-blasted and laser-textured: 8 Etched and laser-textured: 4.2

The main findings of the selected studies can be drawn as follows:

- The surfaces of the zirconia were significantly modified after laser irradiation with Yb-doped fiber or Nd:YVO_4_ laser resulting in aligned retentive regions with macro-scale depth values ranging from 50 up to 120 µm [27].
- The average roughness of laser-textured surfaces using Yb-doped fiber laser system was recorded at around 2.4 µm that was significantly higher when compared to the untreated zirconia surfaces (~ 0.2 µm). Nevertheless, the average roughness recorded for laser-textured surfaces using Er,Cr:YSGG (~ 0.83 µm) was quite similar to those recorded for grit-blasted zirconia surfaces (~ 0.9 µm) [26].
- Surfaces of lithium disilicate-reinforced glass ceramics were also modified by laser irradiation although the etching with HF acid resulted in high mean values of roughness [34]. Lithium disilicate-reinforced glass ceramics textured by Er:YAG laser revealed an average roughness of around 3.5 µm while surfaces textured with Nd:YAG revealed an average roughness of 2.69 µm that was quite similar to the roughness values recorded for etched surfaces (2.64 µm) [34].
- Laser-textured zirconia surfaces showed high values of shear bond strength (SBS) to resin-matrix cements [8, 27]. The SBS values of zirconia surfaces textured on Nd:YVO_4_ laser were slightly higher (~ 33.5 MPa) than those recorded for grit-blasted zirconia surfaces (28 MPa) [27]. Laser-textured zirconia surfaces on CO_2_ laser revealed the highest SBS values (18 ±0.8 MPa) while lowest values (9.1 ± 0.56 MPa) were recorded for untreated zirconia surfaces [8].
- On lithium disilicate-reinforced glass ceramics, higher values of SBS to resin-matrix cements were recorded for specimens textured with a combination of fractional CO_2_ laser irradiation and HF acid etching (~ 22–24 MPa) when compared with grit-blasted specimens (12.2 MPa) [35]. Another study revealed SBS values at around 27.5 MPa for Er:YAG-textured lithium disilicate-reinforced glass ceramics to resin-matrix cements [33, 34]. Surfaces grit-blasted with Al_2_O_3_ particles showed the lowest mean bond strength to the resin composite (15.62 MPa) [33].

## Discussion

This review reported relevant findings on laser-textured lithium disilicate-reinforced glass ceramic or zirconia surfaces and their adhesion to resin-matrix cements. Results showed an increased roughness of the glass ceramics and zirconia when compared to untreated surfaces. Thus, high values of roughness reveal an increase in the retentive area to silane agents, adhesive systems, and resin-matrix cements. Indeed, those findings validate the hypothesis of the present review. Nevertheless, different types of laser and irradiation parameters are reported in literature. A detailed discussion on laser parameters as well as on zirconia and glass ceramics and their adhesion to resin-matrix cements is given as follows.

Previous studies revealed the surface modification of zirconia and glass ceramics by using different laser types (Er,Cr:YSGG, Er:YAG, Nd:YAG, CO_2_, and Nd:YVO_4_) and irradiation parameters (Table 1). A previous study reported adhesive effects of Y-TZP textured by CO_2_ laser irradiation (wavelength of 10.6 mm) at a pulse repetition rate of 1000 Hz and pulse duration of 160 ms at an average power setting of 3 W and at 1 mm away from the surface [8]. Specimens were bonded to a resin-matrix cement and submitted to SBS testing. The highest shear bond strength (SBS) values (1. ±0.8 MPa) were recorded for laser-textured zirconia surfaces while the lowest values (9 ±0.5 MPa) were recorded for untreated zirconia (control group) surfaces [8].

In another study, conical spots were shaped onto zirconia structures by CO_2_ laser texturing and compared to machined, grit-blasted, and polished zirconia surfaces [29]. The morphological aspects of the surfaces were inspected by scanning electron microscopy (SEM) while the zirconia phases were identified by X-ray diffraction (XRD). Surfaces were bonded to a resin-matrix cements and assessed by shear bond strength assays. The shear bond strength of grit-blasted or laser-textured zirconia to resin cement was 2.3 times higher than that for untreated zirconia. Surface modification by laser spotting improved the SBS to the resin cement. However, no significant difference was detected between grit-blasted and laser-textured surfaces [32].

In a previous study, a texturing on Y-TZP resin at different laser beam incidence angles (90, 75, 60, and 45 degree) was assessed using a femtosecond laser, and the SBS values of Y-TZP to resin cement (Rely X™, 3 M ESPE, USA) were measured [31]. The SBS values of each specimen was measured using a universal testing machine at crosshead speed of 1 mm/min. SEM images of the zirconia to resin cement interfaces showed the profile of the orifices shaped by the femtosecond laser. A higher contact area between Y-TZP and resin-matrix cement increased as function of the decrease in the laser beam incidence, as seen in Table 1. The results showed that the laser beam was applied to a surface at an angle of 45 degree which resulted in significantly higher SBS (~ 18 MPa) than that recorded for other groups: at 90 degree (~ 10. MPa), at 75 degree (~ 13 MPa), and at 60 degree (~ 15 MPa). Thus, findings revealed that the decrease of the angle between the ceramic surface and the laser beam resulted in an increase in the contact area of Y-TZP and resin-matrix cement leading to higher SBS values [31]. A femtosecond laser irradiation was also assessed on highly translucent zirconia or lithium disilicate-reinforced glass ceramics producing dot patterns with a line distance of 20 or 40 µm over the surfaces. Dot patterns were produced with peak fluence of 4 or 8 J/cm^2^ and irradiation number of 10 or 20 shots. Roughness parameters were measured by 3D confocal laser microscopy, and the microstructure was analyzed by SEM. The findings revealed similar morphological aspects for both glass ceramic and zirconia considering the surface modification parameters. The laser-textured surfaces with crossed-line patterns at a distance of 20 or 40 µm revealed a great potential for further studies [17].

A previous study also assessed the effects of thermal cycling on the SBS values of laser-textured zirconia surfaces to resin-matrix cements. Y-TZP was textured on Yb-doped laser irradiation at a pulse repetition rate of 60 Hz and pulse duration of 6.6 s and at an average power setting of 24 W [26]. Then, laser-textured surfaces were conditioned with a primer adhesive and bonded to a resin-matrix cement (Panavia V5™, Kuraray, Japan) [26]. Half of the specimens were submitted to thermal cycling, and then, the specimens were assessed by SBS testing. After thermal cycling, laser-textured Y-TZP surfaces to the resin-matrix cement were slightly higher (10 MPa, respectively) than those recorded for grit-blasted zirconia ones (2 MPa) [23]. There were no significant differences in SBS values when comparing laser-textured (12 MPa) and grit-blasted (14.5 MPa) surfaces without the effect of the thermal cycling [26]. Even though SBS values were not statistically different without thermal cycling, the adhesion of the laser-textured zirconia surfaces reached noticeable high adhesion to resin-matrix cements. In another study, zirconia surfaces were textured on Yb-doped pulsed fiber laser irradiation (wavelength of 1070 nm) with a maximum average output power of 20 W, a pulse duration of 100 ns, and a repetition rate in the range 20–100 kHz [28]. Surfaces were conditioned with a primer adhesive following by bonding to a flowable resin-matrix composite (Tetric Evo Flow™, Ivoclar Vivadent, Liechtenstein) and then assessed by SBS tests [28]. Laser-textured surfaces revealed higher fracture forces to resin-matrix cements (132.5 ±61.35 N) when compared with non-textured ones (105.7 ±56.1 N). However, the difference was not considered statistically significant (*p* = 0.2155).

**Fig. 2:**
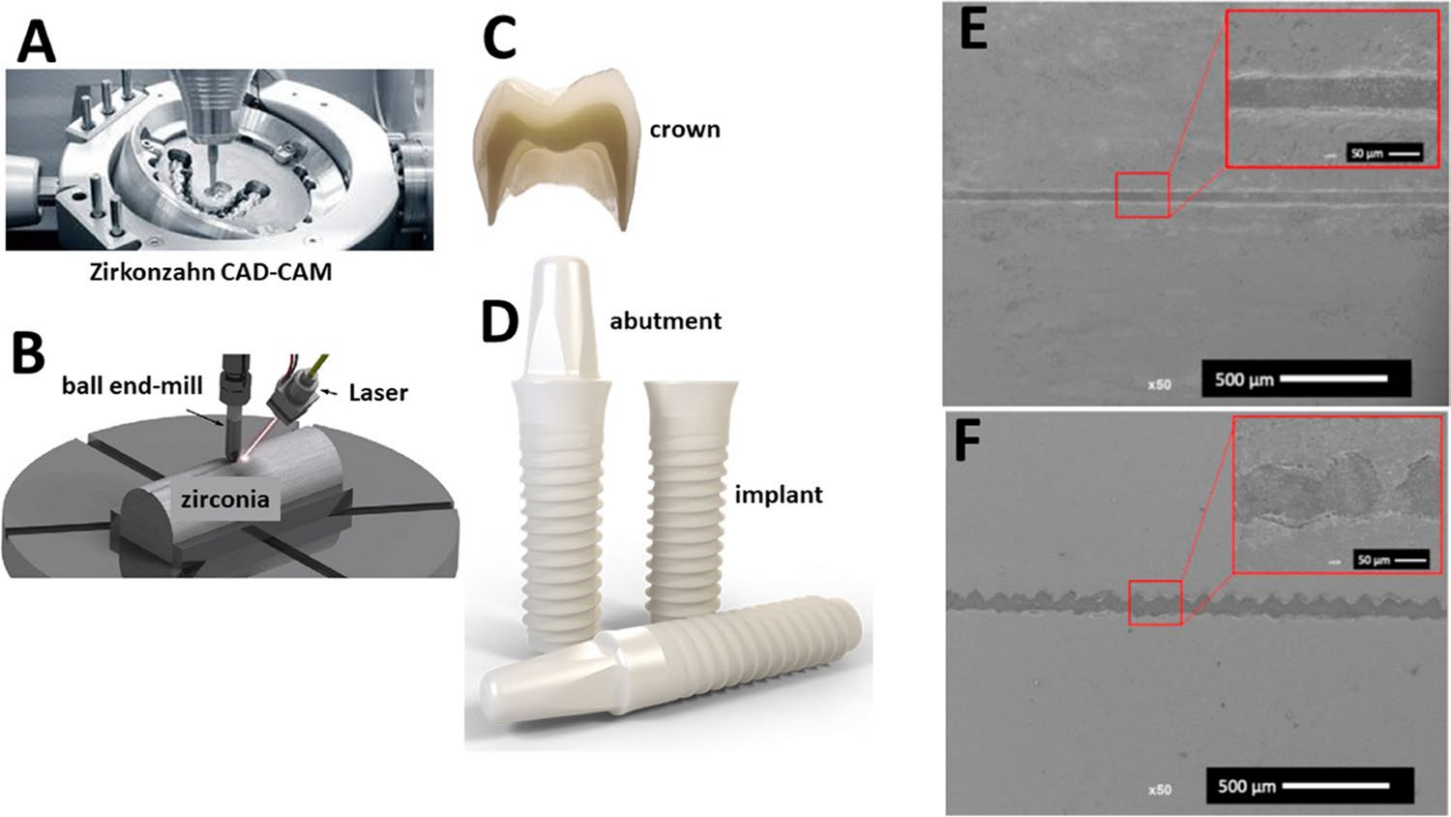
Schematics of the **A** CAD-CAM and **B** laser approaches for **C** crown and **D** implant abutment surfaces. SEM images of the interfaces of **E** non-textured and **F** laser-textured zirconia to a resin-matrix cement. Adapted from Henriques et al. [7]

In a previous selected study, Y-TZP or Ce-TZP surfaces were modified on Nd:YVO_4_ laser irradiation (wavelength of 1065 nm) at 70 kHz from 60 mm away to the surface [27]. Morphological aspects of surfaces showed aligned retentive regions with macro-scale depth values ranging from 50 to 120 µm [27]. The rough profile of the zirconia to the resin-matrix cement interface can be noticed in Fig. 2 while non-textured zirconia surfaces provide a smooth interface profile. Surfaces were conditioned with a primer adhesive (Clearfil Ceramic Primer Plus™, Kuraray, Japan) and bonded to resin-matrix cements (Gradia™, GC, Corp, Japan). Half of the specimens were submitted to thermal cycling, and then, all the specimens were mechanically tested by SBS tests. After thermal cycling, SBS values of laser-textured Y-TZP or Ce-TZP surfaces to the resin-matrix cement were slightly higher (33.5 and 24.5 MPa, respectively) than those recorded for grit-blasted zirconia ones (15.5 and 16.5 MPa, respectively) [27]. There were no significant differences in SBS values when comparing laser-textured Y-TZP or Ce-TZP (28.6 and 27.6 MPa, respectively) and grit-blasted surfaces (28.9 and 25.6 MPa, respectively) without the effect of the thermal cycling [27].

Yttria-stabilized tetragonal zirconia polycrystals (Y-TZP) are produced by introducing 2–5 mol% Y_2_O_3_ into ZrO_2_ formulations [37, 38] for stabilization of the tetragonal phase that results in Y-TZP with a significant increase in the following properties: elastic modulus at 230–270 GPa, fracture toughness of approximately 9–10 MPa.m^1/2^, flexural strength values at around 1200 MPa, and hardness at 1.1 GPa [39, 40]. Also, mechanical properties of zirconia are improved when the tetragonal phase is stabilized by the incorporation of small contents of oxides such as cerium oxide (ceria, CeO_2_), magnesium oxide (magnesia, MgO), and calcium oxide (calcia, CaO) [37, 38]. The abovementioned mechanical property values are significantly higher when compared with those recorded for glass ceramics. Additionally, oxide-stabilized zirconia has an inherent and remarkable pathway to inhibit the propagation of cracks when the material is submitted to high stresses (i.e., from surface modification method). High stresses result in a phase transformation from tetragonal to the monoclinic with an increase in the surrounding volume leading to compressive stresses over the crack. For instance, tetragonal-to-monoclinic phase conversion can take place under cyclic stresses from thermal fluctuations or mastication loading as well as can occur over the surface modification by grit blasting or laser texturing [37–42]. Nevertheless, Y-TZP is susceptible to the propagation of cracks depending on the pressure, medium, and surface modification parameters [41–43]. Thus, zirconia surface modification is still a contemporary challenge considering a balance among chemical stability, physical properties, and degradation behavior. It should be emphasized that the integrity of the zirconia or glass ceramic prosthetic structures can be readily compromised by improper treatment of inner surfaces for cementation and/or failures in the cementation procedures.

Lithium disilicate-reinforced glass ceramic contains approximately 65% volume fraction of lithium disilicate crystals, 34% volume fraction of residual glass, and 1% volume fraction of porosity after heat treatment [44]. The glass matrix is derived from a multi-component system, formulated from SiO_2_-Li_2_O-K_2_O-ZnO-Al_2_O_3_-La_2_O_3_-P_2_O_5_ glass system [44, 46]. Lithium disilicate glass ceramics have shown elastic modulus of around 60–105 GPa, fracture toughness at 2–3 MPa.m^1/2^, bend strength values around 320 ± 30 MPa, and hardness at 5.5 ± 0.3 GPa [44–47]. Such properties are adequate for their application on anterior teeth region although depending on the patient conditions. Thus, low mean values of strength and fracture toughness of the glass ceramics can induce mechanical failures over the surface modification method as well as under cyclic loading in the oral cavity.

In a previous study, lithium disilicate-reinforced glass ceramics were textured on CO_2_ laser irradiation (wavelength of 10.6 µm) at two different irradiation intensities (10W and 28 J or 20 W and 28 J) at a pulse repetition rate of 200 Hz and pulse duration of 1.7 ms or 0.58 ms [35]. Surfaces were conditioned with a silane compound (Silane Bond Enhancer™, Pulpdent Corp., USA) followed by bonding to a resin-matrix cement (Clearfill SA Luting™, Kuraray, Japan), and then, the specimens were submitted to SBS tests [35]. The highest SBS values to resin-matrix cement were recorded for specimens textured with a combination of fractional CO_2_ laser irradiation and HF acid etching (22.4 and 24.3 MPa, respectively). Grit-blasted specimens exhibited the lowest shear bond strength (12.2 MPa) to resin-matrix cements [35]. Considering the glass matrix of lithium disilicate-reinforced glass ceramics, roughness can be increased by etching with 5–10% HF for further adhesion to resin-matrix cements. Additionally, a combination with laser texturing approach becomes beneficial for increasing the retentive area leading to a high bonding to the resin-matrix cement.

Another previous study reported the influence of grit blasting, acid etching, and laser irradiation on the shear bond strength of lithium disilicate-reinforced glass ceramic (IPS Empress 2™, Ivovlar Vivadent, Liechcheinstein) and a feldspar-based porcelain (VITA VM 9™, VITA Zahnfabrik, Germany) to a resin-matrix cement (Panavia 2.0, Kuraray, Japan) [36]. Each of the two ceramic groups was further divided into five groups regarding the surface treatment method and parameters. The laser-textured surfaces were irradiated with Er:YAG laser at 1 mm distance, 500 mJ energy, 100 μs pulse length, 20 Hz, and 10 W power. After bonding to the resin-matrix cement, specimens were stored in water for 24 h and then assessed by SBS test using a universal testing machine at a crosshead speed of 0.5 mm/min. Statistical analyses indicated that the SBS was significantly affected by the surface modification methods (*p* < 0.05), although there was significant correlation between the ceramic systems. Grit-blasted plus laser-textured surfaces showed the highest mean SBS values for both ceramics groups. Atomic force microscopy images revealed a rough morphological aspect for grit-blasted surfaces [36].

In another study, lithium disilicate-reinforced glass ceramic was also modified by Er:YAG (200 mJ, pulse repletion at 10 pps) or Nd:YAG (120 mJ, pulse repletion at 15 pps) pulsed laser irradiation [34]. Groups of specimens were etched with HF, and all of the surfaces were conditioned with silane coating (Monobond Plus™, Ivoclar Vivadent, Liechtenstein) and then bonded to a dual-cure resin-matrix cement (Variolink II™, Ivoclar Vivadent, Liechtenstein). Lithium disilicate-reinforced glass ceramics textured by Er:YAG laser revealed an average roughness of around 3.5 µm while surfaces textured by Nd:YAG laser revealed an average roughness of 2.69 µm that was quite similar to the roughness values recorded for etched surfaces (2.64 µm) [31]. Er:YAG-textured surfaces revealed the highest SBS values to resin-matrix cements (27.5 MPa). Thus, lithium disilicate-reinforced glass ceramics etched with HF acid or laser-textured resulted in surfaces with roughness values higher than those recorded for untreated surfaces [34].

Nowadays, ceramics and glass ceramics are used to synthesize metal free (e.g., zirconia or zirconia-to-porcelain systems) and metal ceramic (e.g., feldspar-based ceramic fused on metallic materials) for implant- or teeth-supported prostheses (Fig. 2). On teeth, crowns or multi-unit prostheses are retained by using resin-matrix cements. Screw or cement-retained crowns consist of two ways of retaining the implant abutment prosthetic [1, 48, 49]. Thus, ceramics and glass ceramics have shown optical properties to significantly improve esthetic appearances, although higher failure rates are associated with fractures, crack propagation, and poor cement and/or bonded retention [1, 50–52]. Thus, the brittle mechanical behavior of glass ceramics and zirconia remains a concern regarding catastrophic fracture or abrupt stress distribution across the structural materials and interfaces. High concentration of stresses at prosthetic structural materials can increase the risks of brittle fractures at the zirconia or glass ceramics to adhesive and resin-matrix interfaces [1, 7, 41–44]. Then, surface modification of ceramics using traditional physical methods and laser-texturing approaches can be optimized regarding novel approaches to avoid clinical failures.

Even though a limited number of studies in literature, the selected studies reveal noteworthy data on laser-textured lithium disilicate-reinforced glass ceramic or zirconia surfaces and their adhesion to resin-matrix cements. In fact, several laser protocols have been assessed to enhance the retentive morphological aspects and increase the roughness for adhesion of ceramics and glass ceramics to resin-matrix cements. Indeed, the variability of laser types and irradiation parameters brings a broad data on the efficiency of the laser-assisted methods for surface modification of zirconia and glass ceramics. Also, further studies are performed to validate the effects of different laser parameters, restorative materials, and types of resin-matrix cements. Laser-assisted methods have been increasingly gathering attention in the technological field although most of laser apparatus are still quite expensive for clinical or laboratory application.

## Conclusions

Within the limitations of the selected studies, the main outcomes can be drawn as follows:

- On lithium disilicate-reinforced glass ceramics, higher shear bond strength values to resin-matrix cements were recorded for specimens textured with a combination of fractional CO_2_ laser irradiation and HF acid etching when compared with grit-blasted specimens. In fact, the association of traditional surface modification methods and laser-texturing provide beneficial effects for adhesion of glass-ceramics to resin-matrix cements.
- Laser-textured zirconia surfaces on CO_2_ or Nd:YVO_4_ laser revealed higher shear bond strength to resin-matrix cements than those recorded for untreated zirconia surfaces. Thus, laser texturing approaches can become an alternative method for the modification of zirconia surfaces.
- The laser irradiation at high power increases the roughness of the inner surface of lithium disilicate-reinforced glass ceramic or zirconia veneers and crowns leading to an enhanced bond strength to resin-matrix cements. Thus, the laser type and irradiation parameters can be adjusted to enhance the macro- and micro-scale retention of zirconia and glass ceramics surfaces. Further studies should consider other parameters related to the power output, frequency, and mode as well as the fluence of the laser irradiation to establish a standard guidelines for laser-texturing.

## Data Availability

Data will be available upon reasonable request.
